# Characterization and discrimination of milk volatiles from donkey fed different roughages using GC-IMS

**DOI:** 10.3389/fnut.2025.1652665

**Published:** 2025-10-14

**Authors:** Xinyi Du, Yan Zhao, Lu Ding, Fei Huang, Wei Ren, Zongjie Ma, Mingxia Zhu, Guiqin Liu, Muhammad Zahoor Khan, Changfa Wang, Miaomiao Zhou, Mengmeng Li

**Affiliations:** School of Agriculture and Biology, Liaocheng Research Institute of Donkey High-Efficiency Breeding and Ecological Feeding, Liaocheng University, Liaocheng, China

**Keywords:** donkeys, roughages, donkey milk, volatile compounds, GC-IMS

## Abstract

**Introduction:**

The flavor is one of the important qualities of milk, which the effect of roughage on the composition of volatile compounds (VOCs) in donkey milk is unclear.

**Methods:**

This study comprehensively analyzed the VOCs of milk from donkeys fed with corn straw (G1), wheat hulls (G2), or wheat straw (G3) using GC-lMS combined with multivariate analysis.

**Results:**

A total of 43 VOCs were identified in donkey milk including 27.91% esters, 25.58% ketones, 18.60% aldehydes, and 16.28% alcohols. The levels of esters and aldehydes were significantly higher in the G1 group than in the G2 and G3 groups, whereas the opposite was true for alkenes. The 13 VOCs with relative odor activity value >1 were the major characteristic flavor compounds of donkey milk, mainly thiocyanic acid methyl ester, acetic acid ethyl ester, and hexanal. The VOCs in the milk from donkeys fed with corn straw (G1), wheat hulls (G2), or wheat straw (G3) were well differentiated using two-dimensional, difference, three dimensional spectra, and fingerprint gallery plots. A total of 23 different VOCs were identified as potential markers to distinguish the milk of donkeys fed with different roughage, including 2-acetylpyrrole, benzaldehyde-dimer, and hexanal-monomer.

**Conclusion:**

Our study indicates that the VOCs in donkey milk are influenced by different roughage fed to donkeys and provided a theoretical basis for the regulation of VOCs in donkey milk by roughages.

## 1 Introduction

As a fundamental nutritional source globally, milk consumption and dairy product utilization have seen unprecedented growth since the beginning of the twenty-first century. As consumers seek dairy products with high nutritional value and diversity, donkey milk has emerged as a notable option. Distinguished by its rich nutritional profile and unique flavor, donkey milk has garnered significant attention in recent years (1). It is recognized as a high-quality dairy product, rich in essential nutrients such as vitamin C, lysozyme, whey proteins, and polyunsaturated fatty acids (PUFAs). Furthermore, donkey milk is associated with several health-promoting properties, including anti-inflammatory, antibacterial, antioxidant, and anti-diabetic effects (2–4). Additionally, donkey milk has a pleasantly creamy, slightly sweet flavor with a long-lasting aftertaste (5). As a result, it is favored by some consumers and has emerged as one of the preferred choices in the dairy market.

Flavor, a complex combination of taste and aroma, plays a crucial role in consumer preference (6). The formation of flavor is influenced by volatile compounds (VOCs), which include aldehydes, ketones, esters, alcohols, acids, hydrocarbons, and nitrogen-containing compounds, among others (7, 8). VOCs in food products arise from both intrinsic odors and chemical reactions during processing, such as Maillard reaction, caramelization, oxidation of lipids, carbohydrate degradation, and degradation of proteins (9). In milk, the primary nutrients—proteins, fats, and carbohydrates—are influenced by various factors including breed, diet, and seasonality (10). Roughage, an important component of herbivore feed, plays a key role in shaping both the composition and flavor of milk (11). For example, a study found that substituting alfalfa with red clover as the sole feed reduced the production of milk, fat, protein, and lactose (12). Similarly, replacing grass silage with corn silage altered the fatty acid composition of milk, decreasing n-3 PUFA concentrations and increasing n-6 PUFA levels, thus raising the n-6/n-3 PUFA ratio (13). The type of feed can influence the sensory attributes of milk, either directly through the transfer of VOCs or indirectly via non-volatile substrates that serve as VOC precursors (14). However, there is limited research on how roughage affects the VOCs of donkey milk.

Gas chromatography-ion mobility spectrometry (GC-IMS) is an advanced method for analyzing VOCs (15), providing benefits like enhanced sensitivity, fast detection, ease in operation, and cost-effectiveness. Moreover, in food flavor analysis, it is also considered a powerful tool (16). Recent studies have employed GC-IMS to examine VOCs in raw cow milk from different regions in China (8). Additionally, principal component analysis (PCA) in combination with GC-IMS has been employed to identify VOCs associated with the spoilage of yak, cattle-yak, and cow milk during refrigeration (17). Furthermore, GC-IMS and chemometric analysis have been used to differentiate VOC profiles between cow milk powder and powders from horse, donkey and camel milk (18). The impact of different drying methods on VOCs in yak milk powder has also been investigated using GC-IMS and PCA, comparing spray drying and freeze-drying techniques (19). Building upon these studies, the present work aims to examine the influence of roughage on the VOC profile of donkey milk, employing GC-IMS and multivariate statistical methods. The findings of this study will enhance the understanding of how roughage influences the aroma profile of donkey milk, offering a theoretical basis for regulating VOCs in donkey milk through dietary interventions.

## 2 Materials and methods

### 2.1 Sample collection

The Institutional Animal Care and Use Committee of Liaocheng University approved all animal experiments under approval number: 2023022706. Raw milk samples were obtained from 27 healthy, prolific female donkeys, 5 years of age (60 ± 15 days into lactation and 286 ± 25 kg body weight). The female donkeys were randomly divided into three groups (9 donkeys in each group) and fed three different types of roughage. The approximate composition of the three types of roughage—corn straw, wheat hulls and wheat straw—are shown in Table 1. The dry matter was determined by direct drying method. The moisture in the diet was evaporated in the drying oven, and then the dry matter content was calculated. Crude protein was determined by Kjeldahl method. Crude fat was determined by oil gravimetric method. The determination of crude ash is to obtain the residue after the sample is burned at 550 °C. The residue is expressed by mass fraction, that is, the content of crude ash in the sample. Neutral detergent fiber and acid detergent fiber were determined by detergent fiber analysis. Calcium was determined by potassium permanganate titration. Phosphorus was determined by molybdenum yellow spectrophotometry. The basic dietary formulation of the diets was as follows: 80.00% roughage (G1: Corn straw; G2: Wheat wells; G3: Wheat straw), 15.50% corn, 3.10% soybean, 1.20% premix and 0.20% salt. The approximate analysis of the diets was as follows: G1 (dry matter: 95.49%, crush ash: 11.50%, crush protein: 6.30%, crush fat: 2.66%), G2 (dry matter: 93.53%, crush ash: 11.14%, crush protein: 6.50%, crush fat: 2.33%), G3 (dry matter: 91.48%, crude ash: 12.06%, crude protein: 6.12%, crude fat: 2.31%). Except for the roughage, the other feed components were the same, and the donkeys had free access to water. The Donkeys were housed in the same semi-dense system and fed twice a day (09:00, 16:00). The experiment lasted 6 weeks, with the first 2 weeks as the study period and the last 4 weeks as the formal period. At the end of the study, milk samples were collected from each donkey (10:00). The milk was immediately frozen and maintained at −80 °C until analysis using GC-IMS.

**Table 1:** Roughage proximate composition.

**Item**	**Corn straw**	**Wheat hulls**	**Wheat straw**
Dry matter (%)	93.70	92.58	89.92
Crude protein (% DM)	4.28	5.08	4.82
Crude fat (% DM)	1.90	1.27	1.02
Crude ash (% DM)	7.48	7.03	9.21
Neutral detergent fiber (% DM)	68.30	55.81	62.32
Acid detergent fiber (% DM)	36.60	34.98	36.28
Calcium (% DM)	0.57	0.34	0.31
Phosphorus (% DM)	0.18	0.24	0.20

DM, dry matter.

### 2.2 GC-IMS

The analysis of VOCs present in donkey milk was conducted using FlavorSpec^®^ (Gesellschaft für Analytische Sensorysteme GmbH, G.A.S., Dortmund, Germany) equipped with an autosampler unit (CTC-PAL, CTC Analytics AG, Zwingen, Switzerland). Nothing is added to the blank sample. Donkey milk samples were thawed at 4 °C. A 5 mL sample of donkey milk was placed in a 20 mL headspace glass bottle and incubated at 60 °C for 15 min while rotating at 500 rpm. Then, 500 μL of headspace gas was automatically injected into a heated injector set at 85 °C. The temperature of the gas chromatography column was set to 40 °C, and nitrogen gas (purity ≥ 99.999%) was used to drive the headspace to the capillary column (MXT-5, 15 m × 0.53 mm × 1.0 μm). The carrier gas flow program was as follows: increase to 2 mL/min within 0–2 min, to 20 mL/min within 2–10 min, and finally to 100 mL/min within 10–20 min. The drift tube of the IMS instrument is 9.8-cm long, with drift temperatures of 60 °C and 45 °C, respectively. The drift tube voltage was set to 5 kV and the drift gas was nitrogen (purity ≥ 99.999%) with a flow rate of 150 mL/min. 3H ionization was performed in positive ion mode.

### 2.3 VOCs analysis

The VOCs data of donkey milk were collected and analyzed using the VOCal instrument analysis software, the GC-IMS library, and built-in plugins (Reporter and Gallery Plot). VOCal, the National Institute of Standards and Technology (NIST), and the built-in GC-IMS database in the software were used for qualitative analysis of VOCs. All samples were analyzed in triplicate. N-ketone C4-C9 (Sinopharm Chemical Reagent Beijing Co., Ltd., China) was used as an external reference to compare the retention indices (RIs) of VOCs of the samples under the same conditions. The Reporter plugin is used to compare spectral differences between samples. The Gallery Plot plugin is used to compare fingerprints of different samples and analyze VOCs between different samples. The key flavor compounds refer to compounds with relative odor activity values (ROVA) ≥ 1. In ROAV calculation, it is not necessary to distinguish haploid and diploid of the same VOCs, and the concentration is directly combined and calculated based on the threshold of monomer. The relative content is calculated by area normalization method. When calculating the peak area or peak area percentage, the level of the compound does not mean its quantity, and the amounts or concentration of the compound cannot be calculated. The ROAVmax of the compound that contributes the most to the aroma components is defined as 100, and the other ROAVs of the remaining components are calculated according to the following formula. The relative content was calculated by area normalization method.


(1) $$\begin{array}{c} \text{ROAVi}=\frac{{\text{C}}_{\text{i}}}{{\text{C}}_{\max}}\times \frac{{\text{T}}_{\text{max}}}{{\text{T}}_{\text{i}}}\times 100 \end{array}$$

*C*_*i*_ represent the relative content (%) of each VOC, relative content (%) = peak area of the substance / total peak area of all volatile substances × 100; *C*_max_ represent the relative content (%) of the compound that contributes the most to VOC content; *T*_*i*_ represent the threshold (μg/kg) in water of each VOC; *T*_max_ represent the aroma threshold (μg/kg) in water of the compound that contributes the most to VOC content.

### 2.4 Statistical analysis

SPSS version 21.0 (SPSS Inc., Chicago, IL, USA) was used for data processing. One-way analysis of variance (ANOVA) followed by Tukey's test was performed for comparisons. Results are expressed as mean ± standard error of mean (SEM, *n* = 9), with significance set at *P* < 0.05. The differential VOCs were identified using criteria of variable importance in projection (VIP) > 1 and *P* < 0.05. GraphPad Prism 9.0 (Graph-Pad Software, Inc.) was used to visualize data statistical results, while MetaboAnalyst 5.0 (https://www.metaboanalyst.ca/) online software was used for orthogonal partial least squares discriminant analysis (OPLS-DA), and heatmap analysis. In metaboanalyst, the study used sum normalization, logarithmic transformation and Pareto scaling for data preprocessing. The relative intensity after the above standardized steps is standardized intensity, which is used to ensure that the comparison of VOCs differences between different dietary groups is fair and repeatable.

## 3 Results

### 3.1 VOC profiles

**Figure 1:**
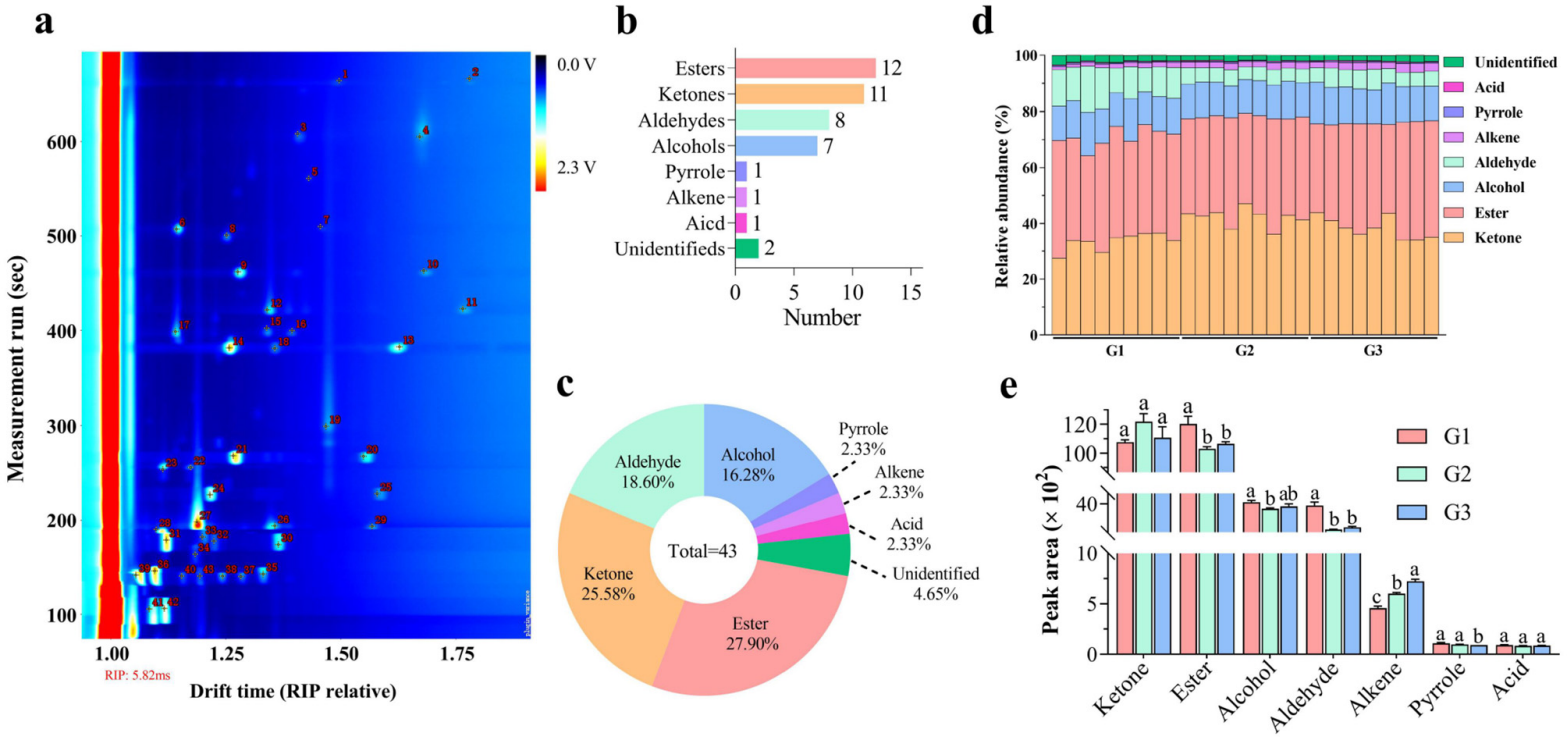
VOC profiles of different donkey milks. Number of VOCs **(a)**. Number **(b)** and percentage **(c)** of VOC categories. Relative abundance **(d)** and concentration **(e)** of VOC classes in donkey milk. Data presented as mean ± standard error of mean (SEM) (*n* = 9), where different letters indicate significant difference (*P* < 0.05). RIP, reactive ion peak; G1, corn straw; G2, wheat hulls; G3, wheat straw.

A total of 43 VOCs were detected in donkey milk, as illustrated in Figure 1a and detailed in Table 2. These VOCs were categorized into eight groups: 12 esters, 11 ketones, 8 aldehydes, 7 alcohols, 1 pyrrole, 1 alkene, 1 acid, and 2 unidentified compounds, accounting for 27.91%, 25.58%, 18.60%, 16.28%, 2.33%, 2.33%, 2.33%, and 4.65% of the total, respectively (Figures 1b, c). Ketones and esters were the most prevalent VOCs, followed by alcohols and aldehydes (Figure 1d). Significantly higher levels of esters and aldehydes were found in the G1 group compared to both G2 and G3 groups (*P* < 0.05 and *P* < 0.001, respectively). The alcohols level was significantly higher in G1 group than G2 group (*P* < 0.05). In contrast, alkene levels were significantly elevated in the G3 group relative to both the G1 and G2 groups, with the G2 group also exhibiting significantly higher alkene levels than the G1 group (*P* < 0.001). Additionally, the pyrrole level was significantly reduced in the G3 group compared to both the G1 and G2 groups (*P* < 0.05).

**Table 2:** Information on VOCs in donkey milk.

**Count**	**Compound**	**Category**	**CAS#**	**Formula**	**MW**	**RI**	**Rt [sec]**	**Dt [a.u.]**	**Comment**
1	2-acetylpyrrole	Pyrrole	C1072839	C6H7NO	109.1	1,054.7	663.335	1.49759	
2	Heptanoic acid	Acid	C111148	C7H14O2	130.2	1,056.0	665.588	1.78087	
3	Acetic acid, hexyl ester	Esters	C142927	C8H16O2	144.2	1,018.5	607.646	1.40741	
4	1-phellandrene	Alkene	C4221981	C10H16	136.2	1,016.4	604.599	1.67324	
5	2-octanol	Alcohols	C123966	C8H18O	130.2	989.5	560.688	1.43168	
6	Benzaldehyde-M	Aldehydes	C100527	C7H6O	106.1	963.3	506.898	1.14466	Monomer
7	Benzaldehyde-D	Aldehydes	C100527	C7H6O	106.1	964.8	509.745	1.45735	Dimer
8	(E)-hept-2-enal	Aldehydes	C18829555	C7H12O	112.2	960.1	500.635	1.25383	
9	5-methyl-3-heptanone-M	Ketones	C541855	C8H16O	128.2	938.9	461.351	1.27809	Monomer
10	5-methyl-3-heptanone-D	Ketones	C541855	C8H16O	128.2	939.5	462.490	1.68108	Dimer
11	Amyl acetate-D	Esters	C628637	C7H14O2	130.2	916.2	422.636	1.76599	Dimer
12	Amyl acetate-M	Esters	C628637	C7H14O2	130.2	915.5	421.497	1.34144	Monomer
13	2-heptanone-D	Ketones	C110430	C7H14O	114.2	890.1	382.213	1.62852	Dimer
14	2-heptanone-M	Ketones	C110430	C7H14O	114.2	889.3	381.074	1.25923	Monomer
15	Heptanal	Aldehydes	C111717	C7H14O	114.2	903.3	402.140	1.34009	
16	Heptan-2-ol	Alcohols	C543497	C7H16O	116.2	901.5	399.441	1.39591	
17	(Z)-4-heptenal	Aldehydes	C6728310	C7H12O	112.2	900.8	398.340	1.14201	
18	Allyl isothiocyanate	Esters	C57067	C4H5NS	99.2	889.1	380.717	1.35764	
19	Isopropyl methylphosphonofluoridate	Esters	C107448	C4H10FO2P	140.1	825.9	298.866	1.46910	
20	Hexanal-D	Aldehydes	C66251	C6H12O	100.2	796.7	267.248	1.55045	Dimer
21	Hexanal-M	Aldehydes	C66251	C6H12O	100.2	797.3	267.800	1.26811	Monomer
22	3-hexanone	Ketones	C589388	C6H12O	100.2	785.2	255.679	1.17568	
23	Cyclopentanone	Ketones	C120923	C5H8O	84.1	782.9	253.490	1.11443	
24	Ethyl 2-methylpropanoate-M	Esters	C97621	C6H12O2	116.2	752.5	226.666	1.21688	Monomer
25	Ethyl 2-methylpropanoate-D	Esters	C97621	C6H12O2	116.2	753.8	227.761	1.57988	Dimer
26	Thiocyanic acid, methyl ester-D	Esters	C556649	C2H3NS	73.1	709.7	193.548	1.35829	Dimer
27	Thiocyanic acid, methyl ester-M	Esters	C556649	C2H3NS	73.1	715.3	197.594	1.18893	Monomer
28	Eethyl acrylate	Esters	C140885	C5H8O2	100.1	703.8	189.371	1.10106	
29	Unidentified 1	Unidentifieds	-	-	-	708.7	192.829	1.56969	
30	2-pentanone-D	Ketones	C107879	C5H10O	86.1	676.6	173.760	1.36558	Dimer
31	2-pentanone-M	Ketones	C107879	C5H10O	86.1	687.2	178.440	1.12246	Monomer
32	3-methyl-2-butanol	Alcohols	C598754	C5H12O	88.1	684.3	177.140	1.22574	
33	Pentan-2-ol	Alcohols	C6032297	C5H12O	88.1	692.4	181.561	1.20106	
34	1-butanol	Alcohols	C71363	C4H10O	74.1	652.6	163.618	1.18553	
35	Acetic acid ethyl ester-D	Esters	C141786	C4H8O2	88.1	596.9	142.295	1.33268	Dimer
36	Acetic acid ethyl ester-M	Esters	C141786	C4H8O2	88.1	606.2	145.676	1.09687	Monomer
37	Butanal	Aldehydes	C123728	C4H8O	72.1	590.2	139.955	1.28424	
38	2-butanone-D	Ketones	C78933	C4H8O	72.1	591.1	140.271	1.24525	Dimer
39	2-butanone-M	Ketones	C78933	C4H8O	72.1	594.7	141.552	1.05669	Monomer
40	2-propanethiol	Alcohols	C75332	C3H8S	76.2	590.7	140.115	1.15548	
41	Isopropyl alcohol	Alcohols	C67630	C3H8O	60.1	478.4	105.776	1.08488	
42	Acetone	Ketones	C67641	C3H6O	58.1	479.0	105.920	1.11856	
43	Unidentified 2	Unidentifieds	-	-	-	591.8	140.514	1.19484	

The VOCs were identified by retention index (RI) and driff time (Dt). MW, molecular weight; Rt, retention time.

### 3.2 Characteristic VOCs in donkey milk

**Figure 2:**
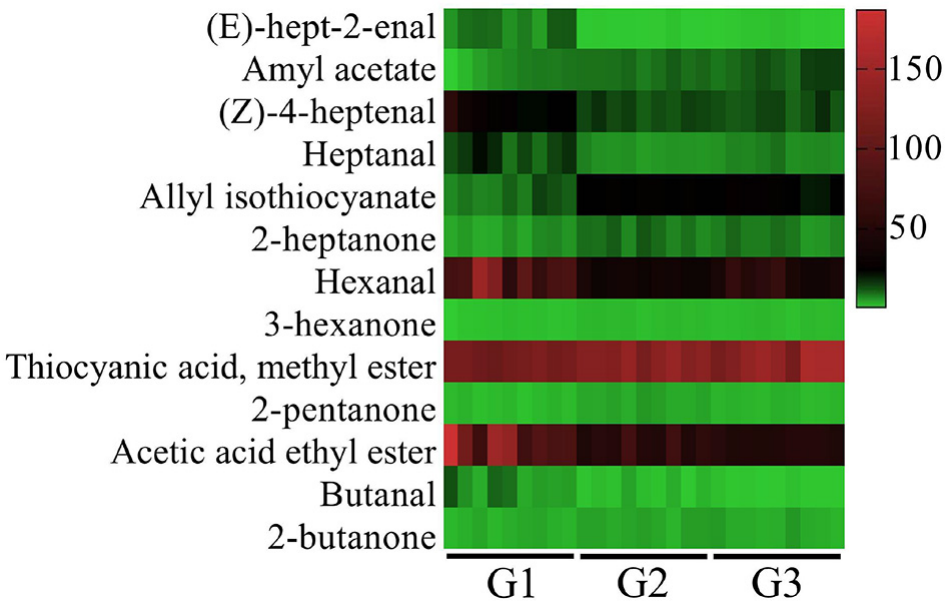
Relative odor activity value (ROAVs ≥ 1) of VOCs in donkey milk. G1, corn straw; G2, wheat hulls; G3, wheat straw.

Thirteen characteristic VOCs with ROAVs greater than 1 were identified in donkey milk, as shown in Figure 2. These compounds included (E)-hept-2-enal, amyl acetate, (Z)-4-heptenal, heptanal, allyl isothiocyanate, 2-heptanone, hexanal, 3-hexanone, thiocyanic acid methyl ester, 2-pentanone, acetic acid ethyl ester, butanal, 2-butanone. The identified VOCs were classified into three categories: five aldehydes, four esters, and four ketones. Among these, Thiocyanic acid methyl ester, acetic acid ethyl ester, and hexanal were found to make the most significant contributions to the overall flavor profile of donkey milk. Additionally, the ROAVs of (E)-hept-2-enal, (Z)-4-heptenal, heptanal, acetic acid ethyl ester and butanal in G2 and G3 groups were significantly lower than those in G1 group, whereas the opposite was true for allyl isothiocyanate, 2-heptanone, hexanal, 3-hexanone and thiocyanic acid methyl ester (*P* < 0.001). The ROAVs of 2-pentanone (*P* < 0.001) and 2-butanone (*P* < 0.05) in G2 group were significantly lower than that in G1 and G3 groups. The ROAV of amyl acetate in group G3 was greater than that in groups G2 and G1, and the ROAV in group G2 was greater than that in group G1 (*P* < 0.001).

### 3.3 Comparison of VOCs

**Figure 3:**
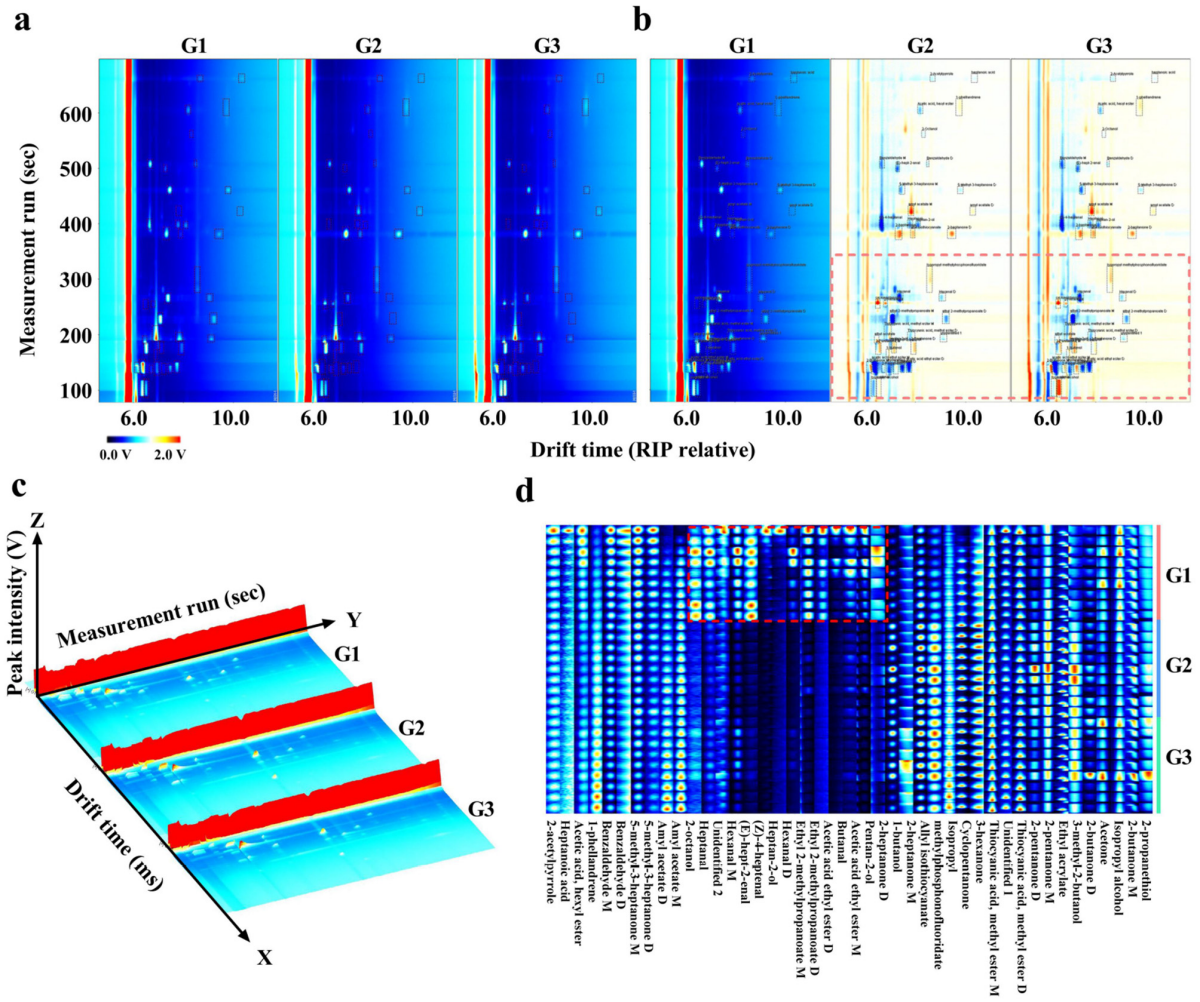
Comparison of VOCs of three types of donkey milk. Two-dimensional **(a)**, difference **(b)**, three-dimensional spectra **(c)** and fingerprint **(d)** of VOCs in donkey milk samples. G1, corn straw; G2, wheat hulls; G3, wheat straw.

As shown in Figure 3, the VOC profiles of donkey milk from three distinct groups were visually represented using two-dimensional spectra, difference spectra, and three-dimensional spectra (Figures 3a–c). The two-dimensional spectra of G1 was chosen as the reference, and the two-dimensional spectra of G2 and G3 were subtracted from the reference. Red signifies a concentration greater than the reference, and blue signifies a concentration lower than the reference. The red and blue spots in the differential spectra of G2 and G3 indicate significant differences from G1. Significant differences in the VOCs were observed between each group of donkey milk, as evidenced by the analysis of the fingerprint gallery plots (Figure 3d). Furthermore, compounds such as pentan-2-ol, acetic acid ethyl ester-monomer, butanal, acetic acid ethyl ester-dimer, ethyl 2-methylpropanoate-dimer, ethyl 2-methylpropanoate-monomer, hexanal-dimer, heptan-2-ol, (Z)-4-heptenal, (E)-hept-2-enal, hexanal-monomer, heptanal, and 2-octanol exhibited distinct signals, indicating the presence of differential components in the fingerprint regions of the G1 group compared to the other two groups (Figure 3d).

### 3.4 Multivariate analysis of VOCs

**Figure 4:**
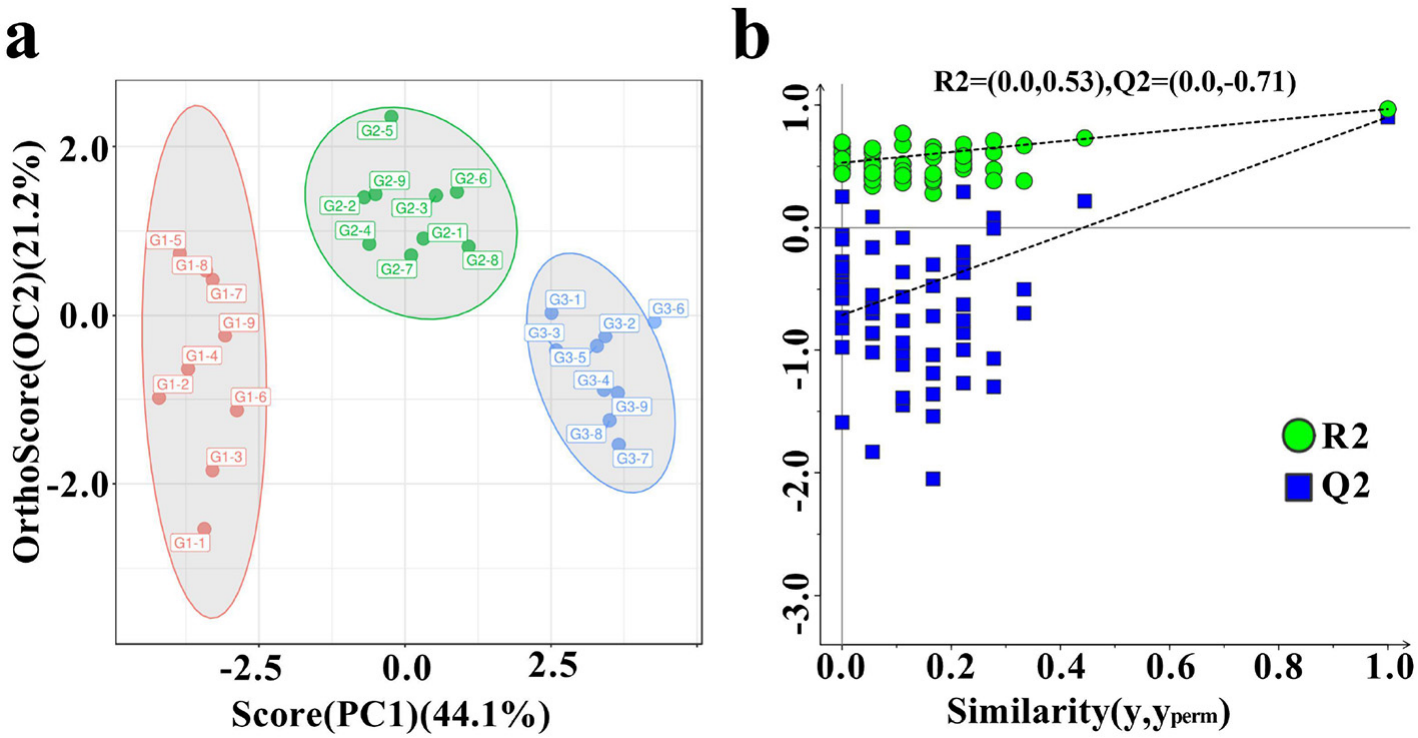
Differences in VOCs in three types of donkey milk. Orthogonal partial least squares discriminant analysis (OPLS-DA) score plot **(a)** and corresponding OPLS–DA validation plot **(b)** of donkey milk VOCs data. G1, corn straw; G2, wheat hulls; G3, wheat straw.

As shown in Figure 4, the donkey milk samples were effectively discriminated using OPLS-DA (Figure 4a). The OPLS-DA model demonstrated robustness, accuracy, and no overfitting, with satisfactory validation results (Figure 4b). The VIP analysis of the OPLS-DA model, presented in Table 3, identified 23 distinct VOCs with VIP values greater than 1 across the three groups of donkey milk. These VOCs were classified into six categories: 9 esters, 8 aldehydes, 2 alcohols, 2 ketones, 1 alkene, and 1 pyrrole (Table 3). A comparison of the VOC levels revealed that the concentrations of (E)-hept-2-enal, (Z)-4-heptenal, 2-octanol, 5-methyl-3-heptanone-dimer, 5-methyl-3-heptanone-monomer, acetic acid, hexyl ester, benzaldehyde-monomer, heptanal, hexanal-dimer, acetic acid ethyl ester-monomer, butanal, ethyl 2-methylpropanoate-dimer, and pentan-2-ol were significantly lower in groups G2 and G3 compared to group G1 (*P* < 0.05). Furthermore, significantly higher levels of 1-phellandrene, thiocyanic acid methyl ester-monomer, and allyl isothiocyanate were found in G2 and G3 groups compared to G1Additionally, the levels of 2-acetylpyrrole, benzaldehyde-dimer, acetic acid ethyl ester-dimer, and ethyl 2-methylpropanoate-monomer were significantly higher in G1 than in G2 and G3, with the G2 group showing higher levels than G3 (*P* < 0.05). The levels of isopropyl methylphosphonofluoridate and amyl acetate-dimer were significantly elevated in G3 compared to G1 and G2 (*P* < 0.05), with G2 showing higher levels than G1. The hexanal-monomer levels were significantly higher in G1 compared to G2 and G3, and G3 exhibited significantly higher levels than G2 (*P* < 0.05).

**Table 3:** Difference of VOCs of donkey meat fed with three different diets (standardized intensity).

**No**.	**Compound**	**G1**	**G2**	**G3**	***P*-value**	**VIP**
1	(E)-hept-2-enal	278.29 ± 22.92^a^	55.17 ± 1.38^b^	58.62 ± 4.54^b^	0.0000	1.342
2	(Z)-4-heptenal	284.19 ± 79.07^a^	98.57 ± 2.86^b^	93.46 ± 2.93^b^	0.0016	1.128
3	1-phellandrene	457.73 ± 20.45^a^	601.47 ± 9.88^b^	723.60 3.20.54^b^	0.0000	1.439
4	2-acetylpyrrole	110.28 ± 5.71^a^	98.81 ± 1.44^b^	91.55 ± 0.89^c^	0.0005	1.017
5	2-octanol	92.66 ± 7.29^a^	33.68 ± 2.15^b^	38.47 ± 2.54^b^	0.0000	1.178
6	5-methyl-3-heptanone D	157.98 ± 9.03^a^	120.32 ± 5.90^b^	104.85 ± 2.48^b^	0.0000	1.292
7	5-methyl-3-heptanone M	900.17 ± 40.08^a^	752.16 ± 27.45^b^	628.41 ± 19.99^b^	0.0000	1.324
8	Acetic acid, hexyl ester	300.48 ± 20.66^a^	187.33 ± 3.90^b^	210.06 ± 5.18^b^	0.0000	1.019
9	Benzaldehyde D	67.22 ± 2.53^a^	49.36 ± 1.41^c^	46.31 ± 0.58^b^	0.0000	1.352
10	Benzaldehyde M	701.08 ± 37.20^a^	512.56 ± 9.39^b^	453.68 ± 4.30^b^	0.0000	1.390
11	Heptanal	326.26 ± 25.18^a^	141.23 ± 4.59^b^	162.78 ± 7.03^b^	0.0000	1.173
12	Hexanal M	1582.74 ± 182.10^c^	427.07 ± 22.06^b^	673.67 ± 78.34^a^	0.0000	1.020
13	Hexanal D	327.25 ± 74.95^a^	88.69 ± 2.70^b^	114.58 ± 8.23^b^	0.0001	1.023
14	Isopropyl methylphosphonofluoridate	1024.40 2448.26^c^	1233.53 ± 13.19^b^	1412.36 ± 35.58^a^	0.0001	1.334
15	Thiocyanic acid, methyl ester M	4215.30 1569.36^b^	4413.19 ± 53.04^a^	4631.22 ± 82.14^a^	0.0009	1.164
16	Acetic acid ethyl ester D	417.27 ± 112.89^c^	145.85 ± 6.16^b^	144.30 4.3.77^a^	0.0138	1.063
17	Acetic acid ethyl ester M	2022.80 22288.32^a^	811.01 ± 57.33^b^	654.74 ± 40.53^b^	0.0000	1.356
18	Allyl isothiocyanate	188.22 ± 11.25^a^	387.98 ± 21.31^b^	370.23 ± 23.59^b^	0.0000	1.190
19	Amyl acetate D	126.06 ± 11.58^c^	160.75 ± 4.80^b^	202.46 ± 8.24^a^	0.0003	1.197
20	Butanal	278.41 ± 43.70^a^	106.94 ± 10.01^b^	76.20 .25.73^b^	0.0000	1.383
21	Ethyl 2-methylpropanoate D	336.09 ± 32.19^a^	148.80 8.3.89^b^	143.73 ± 3.33^b^	0.0000	1.332
22	Ethyl 2-methylpropanoate M	1324.89 ± 100.97^a^	382.52 ± 34.69^b^	311.02 ± 5.49^c^	0.0000	1.419
23	Pentan-2-ol	272.89 ± 34.42^a^	157.44 ± 7.41^b^	142.62 ± 7.09^b^	0.0000	1.103

G1, corn straw; G2, wheat hulls; G3, wheat straw; VIP, variable importance in projection. a–c: Values within the same row that bear different letters differ significantly, whereas those sharing any letter are not significantly different.

## 4 Discussion

Flavor is a key determinant of consumer preferences, representing one of the fundamental characteristics of milk quality. It is primarily defined by the specific composition of VOCs, which contribute significantly to its sensory profile (20, 21). The main VOCs that contribute to the unique flavor of milk include alcohols, aldehydes, ketones, and esters (22). Flavoromics technologies, particularly GC-IMS, have emerged as effective tools for detecting VOCs in food. This method offers advantages such as simplicity, high sensitivity, rapid detection, and no sample preparation (19). The VOCs in donkey milk, sourced from animals fed different roughages, were comprehensively characterized and compared using GC-IMS. A total of 43 VOCs were identified in donkey milk, consistent with the 34 VOCs reported in yak milk from various regions of Gannan using GC-IMS (6). The lower number of VOCs in donkey milk can be attributed to the reduced moisture content of whole milk powder, which typically results in a higher number of VOCs (23). At the same time, VOCs are more abundant in whole milk powder due to the reduced moisture content (24). Additionally, GC-IMS is more adept at detecting low molecular weight compounds compared to GC-MS, which enhances its sensitivity for certain VOCs (25). The predominant VOCs in donkey milk were esters, ketones, aldehydes, and alcohols, with ketones and esters exhibiting the highest concentrations. These findings align with previous GC-IMS studies on donkey milk at various lactation stages (26). Notably, 12 esters were identified in donkey milk, a significantly higher number compared to the 3 esters found in raw cow milk (8). This suggests that esters are particularly abundant in donkey milk. Furthermore, the levels of esters, aldehydes, and pyrroles were significantly higher in milk from donkeys fed corn straw, compared to those fed wheat hulls and wheat straw. The production of esters and aldehydes is closely linked to the free fatty acids present in milk fat, with higher fatty acid levels leading to increased ester and aldehyde production (27). It has also been shown that the lipid composition of roughage influences the lipid profile of donkey milk (28). Higher crude fat content in the diet reduces the proportion of short-chain fatty acids but increases the proportion of monounsaturated and polyunsaturated fatty acids (29).Therefore, the elevated levels of esters and aldehydes in donkey milk may be attributed to the higher crude fat level of the corn straw provided in this study. In this investigation, pyrrole refers exclusively to 2-acetylpyrrole, a condensation product formed between dicarbonyl compounds and ammonia (30). Lactose present in milk serves as a precursor for the generation of α-dicarbonyl compounds (31). A previous study revealed that partial replacement of short alfalfa hay with corn silage increased dry matter intake, which in turn improved milk yield and yields of milk protein and lactose (32). In this study, the elevated level of pyrroles in donkey milk may be attributed to the higher dry matter level of the corn straw provided. Conversely, the concentration of alkenes was lower in milk from donkeys fed corn straw, especially 1-phellandrene. There is a positive correlation between 1-phelandrene in milk and terpene content in botanical composition of pasture (33). The lower level of alkene in donkey milk may be attributed to the lower alkene level of the corn stover provided in this study.

The contribution of each VOC to the overall flavor can be determined using ROAV, and VOCs with ROAV > 1 represents the key flavor compounds (34). In this study, 13 key flavor compounds were identified in donkey milk, including 2-heptanone, hexanal, acetic acid ethyl ester, and butanal. A previous study has similarly identified these compounds as key contributors to the flavor of cow milk (27), suggesting a degree of similarity in flavor characteristics between donkey and cow milk. There seems to be a potential direct transfer of some nonterpene VOCs from feed to milk, as suggested by tentative associations (35). In addition, after Tarantaise cows changed their diet and ingested yarrow, the concentrations of monoterpenes and sesquiterpenes in milk fat gradually increased, and then declining despite continued yarrow intake (36). Therefore, VOCs in dietary components and feeding time may affect the types and contents of VOCs in animal milk, thereby affecting ROAV.

The characteristic VOCs in donkey milk primarily consisted of five aldehydes, four esters, and four ketones. Aldehydes and ketones are particularly notable for their low odor thresholds and are commonly found in dairy products (37). Aldehydes are mainly derived from the Maillard reaction and fatty acid oxidation processes (38). In this study, the fatty aldehydes identified in donkey milk included (E)-hept-2-enal, (Z)-4-heptenal, heptanal, hexanal, and butanal. These compounds, particularly in low concentrations, are associated with green, herbaceous, and grassy aromas (39). Among these, hexanal, which ranked third in terms of ROAV, imparts a grassy, green, and slightly vinegar-like aroma (40). Ester compounds, characterized by low perception thresholds, are crucial to the flavor of food, contributing fruity, floral, and sweet flavors (41). In milk, esters primarily arise from esterification reactions between free fatty acids and alcohols within milk fat (18). In this study, key ester compounds identified in donkey milk included acetyl acetate, allyl isothiocyanate, thiocyanic acid methyl ester, and acetic acid ethyl ester. Notably, acetic acid ethyl ester and thiocyanic acid methyl ester were the most influential in the flavor profile of donkey milk. Acetic acid ethyl ester, which has a high perception threshold, contributes a distinct pineapple smell, while thiocyanic acid methyl ester imparts a garlic aroma (41, 42).

The VOCs differences across samples can be visualized through GC-IMS spectral analyses, including two-dimensional, difference, and three-dimensional spectra. These visualizations facilitate the intuitive and specific comparison of VOC levels between samples, with fingerprints serving as a particularly useful tool for this purpose (19). In the present study, significant spectral differences were observed in donkey milk from animals fed different roughages. This was verified by fingerprint analysis, which revealed notable variations in specific VOCs such as ethyl 2-methylpropanoate, butanal, and acetic acid ethyl ester, particularly in milk from donkeys fed corn straw compared to those fed wheat hulls and wheat straw. The VOCs capable of directly influencing flavor may be absorbed within the digestive tract, particularly in the rumen and or intestine, prior to diffusing into the blood and being conveyed to the mammary gland, or they may enter via the pulmonary route, being inhaled into the lungs, entering the bloodstream, and ultimately diffusing into the mammary gland (36). The rumen microbiota is responsible for the production of odd- and branched-chain fatty acids in milk fat (43). Divergent diets alter rumen fermentation patterns, thereby driving variations in the rumen microbial community structure (44). Feed fermentation hinge on intrinsic feed properties, with wheat straw and wheat hulls showing equal *in vitro* dry-matter disappearance that remains lower than corn stover. Therefore, the properties between wheat straw and wheat husk are similar, while both have significant differences in properties compared to corn straw (45). A study indicated that the low nutrient digestibility of wheat straw may be due to its higher lignin content compared to corn straw, as well as its lower acid detergent fiber content, which is consistent with this study (46). Previous studies have successfully employed fingerprint analysis using GC-IMS to monitor VOC dynamics in dairy products, such as the changes during the ripening of cream cheese (47) and the impact of fatty acid composition on VOCs in pasteurized milk stored at 4 °C (27). Multivariate analysis techniques, including heatmap visualization, PCA, and OPLS-DA, are commonly used to further analyze sample differences (48, 49). For example, the classification of sorghum types has been achieved through GC-IMS combined with multivariate analysis techniques, where OPLS-DA successfully distinguished significant sample variations within varieties (50). In the present study, OPLS-DA effectively distinguished the differences in VOCs across donkey milk samples. The VIP value was used to measure the contribution of each variable to the model classification, with a VIP > 1 indicating a significant contribution (47, 51). Statistically, a *P*-value below the critical threshold of 0.05 typically signifies a significant result, whereas a *P*-value above this threshold suggests a lack of significance (52). A total of 23 different VOCs were identified in donkey milk, with compounds such as ethyl 2-methylpropanoate, butanal, and acetic acid ethyl ester showing consistent results with the fingerprint analysis. These findings indicate that the VOC profile of donkey milk can undergo significant changes depending on the roughage fed to the animals, aligning with previous research on the effects of forage types on milk composition (35).

## 5 Conclusion

Flavor, especially VOCs, is one of the key factors directly affecting consumer choice and acceptance. Some feeds may introduce unpleasant odors or enhance pleasant odors, which in turn guides the optimization of feed formulations and avoids flavor defects in donkey milk. The effects of different types of roughage on VOCs of donkey milk were analyzed and compared using GC-IMS and multivariate analysis. A total of 43 VOCs detected in seven categories. Of these, 13 compounds, including thiocyanic acid methyl ester, acetic acid ethyl ester, and hexanal, were selected as characteristic flavor compounds. Additionally, 23 VOCs were identified as potential markers to differentiate the milk of donkeys fed different types of roughage. The VOCs, particularly esters and aldehydes, in donkey milk were primarily influenced by the type of roughage fed to the donkeys. The outcomes of this study provide insights into how different roughage types influence VOC composition in donkey milk, offering a theoretical foundation for VOC regulation in donkey milk production.

## Data Availability

The original contributions presented in the study are included in the article/supplementary material, further inquiries can be directed to the corresponding author/s.
